# Efficacy and safety of endoscopic cardia constriction ligation with a single-use endoscope versus a reusable endoscope for refractory gastroesophageal reflux disease: protocol for a multicenter randomized controlled trial

**DOI:** 10.3389/fmed.2026.1832310

**Published:** 2026-06-24

**Authors:** Run-hua Li, Ying-ying Yang, Wen Xu, Gui-li Xia, Qing Cheng, Xiao-bing Cui, Ying Zhu, Xiao-hua Bao

**Affiliations:** 1 Department of Gastroenterology, Shenzhen Hospital, Southern Medical University, Shenzhen, Guangdong, China; 2 Shenzhen School of Clinical Medicine, Southern Medical University, Shenzhen, Guangdong, China; 3 Department of Gastroenterology, Daping Hospital, Army Medical University, Chongqing, China; 4 Department of Gastroenterology, No. 964th Hospital, Changchun, China

**Keywords:** disposable endoscopes, endoscopic cardia constriction (ECCL), gastroesophageal reflux disease (GERD), infection control, procedural feasibility and safety, randomized controlled trial, single-use endoscope

## Abstract

**Background and aims:**

Reusable endoscopes require reprocessing and may carry potential concerns regarding cross-contamination, whereas single-use endoscopes may reduce infection-related risks and simplify workflow. However, evidence remains limited on whether single-use endoscopes can provide comparable performance in therapeutic upper gastrointestinal procedures requiring stable visualization, reliable suction, retroflexion, and accessory compatibility. Endoscopic cardia constriction with ligation (ECCL) is a minimally invasive endoscopic treatment for refractory gastroesophageal reflux disease (GERD). This multicenter randomized controlled trial aims to compare the efficacy, safety, and procedural feasibility of ECCL performed with a single-use endoscope versus a reusable endoscope in patients with refractory GERD.

**Methods:**

This is a multicenter, open-label, parallel-group, non-inferiority randomized controlled trial. Ninety-eight adults aged 18–80 years with refractory GERD will be randomized 1:1 to undergo ECCL using either a single-use endoscope or a reusable endoscope. Eligible participants must have typical GERD symptoms for at least 6 months, persistent symptoms despite optimized acid-suppressive therapy, and objective evidence supporting GERD diagnosis. ECCL will be performed according to a standardized protocol. Participants will receive standardized post-procedural acid-suppressive therapy for 2 weeks and will be followed at 3 and 6 months. The primary efficacy endpoint is clinically significant symptom improvement, defined as a decrease in GERD-Q score of ≥4 points from baseline. Change in GERD-Q score will also be analyzed as a continuous supportive outcome. Secondary endpoints include procedural feasibility, safety outcomes, operational stability, device malfunction/defect rates, and acid-suppressive medication use after ECCL, including complete discontinuation of PPI/P-CAB therapy at 3 and 6 months.

**Discussion:**

This trial will provide prospective evidence on whether single-use endoscopes can support ECCL with efficacy, safety, and procedural feasibility comparable to reusable endoscopes. By integrating patient-reported outcomes, standardized procedural assessment, device-related events, and post-procedural medication use, this study may inform future clinical application of single-use endoscopes in therapeutic upper gastrointestinal endoscopy.

**Clinical trial registration:**

ClinicalTrials.gov, identifier NCT07176221.

## 1 Introduction

Reusable endoscopes are widely used in routine clinical practice and require meticulous cleaning and high-level disinfection between procedures. However, concerns remain that reprocessing may not always eliminate the risk of contamination, and the potential for cross-contamination has received increasing attention (1, 2). Single-use endoscopes avoid reprocessing and may reduce infection-related risks while offering performance that is broadly comparable to that of conventional reusable endoscopes (3). However, evidence remains limited regarding whether single-use endoscopes can achieve comparable clinical outcomes and procedural safety in therapeutic upper gastrointestinal interventions that require stable retroflexion, clear visualization, reliable suction, and consistent accessory compatibility.

Gastroesophageal reflux disease (GERD) is a common chronic disorder characterized by typical symptoms, such as heartburn and regurgitation, as well as a range of atypical or extra-esophageal manifestations (4). In China, the prevalence of at least weekly heartburn has been reported to range from 1.9 to 7.0% (5). Persistent GERD can substantially impair quality of life and may increase the risk of Barrett’s esophagus and subsequent neoplastic progression. The pathophysiology of GERD is multifactorial and involves abnormal acid exposure, lower esophageal sphincter (LES) dysfunction, impaired esophageal clearance, hiatal hernia, mucosal barrier injury, inflammatory mediators, and reflux hypersensitivity (6, 7).

Lifestyle modification and acid suppression with proton pump inhibitors (PPIs) (8) or potassium-competitive acid blockers (P-CABs) (9) remain first-line therapies for GERD. Nevertheless, a subset of patients experience refractory GERD symptoms despite optimized medical therapy and may require long-term pharmacologic maintenance. Accumulating evidence suggests that prolonged PPI use may be associated with increased risks of *Clostridioides difficile* infection, community-acquired pneumonia, gastric cancer, and chronic kidney disease (10, 11), while short-term studies have suggested that P-CABs may induce hypergastrinemia (12). In this context, current Chinese expert consensus recommends that, for truly refractory GERD—after careful evaluation to confirm objective reflux and exclude alternative etiologies—endoscopic or surgical intervention may be considered following an individualized risk–benefit assessment (13).

Endoscopic cardia constriction with ligation (ECCL), introduced in 2013 (14), is performed by suctioning and ligating mucosal and underlying tissue above the dentate line to create folds. Subsequent healing with scar formation narrows the cardia and may increase LES pressure, thereby relieving reflux symptoms (15). ECCL is minimally invasive and technically straightforward. Reported complications include transient retrosternal pain and bleeding after band sloughing, whereas serious adverse events appear uncommon in existing reports. Available clinical evidence for ECCL remains preliminary but encouraging. In an early study of endoscopic cardial constriction involving 47 patients with GERD (16), the procedure was successfully completed in all patients, with a mean procedure time of 6.5 min, and no severe bleeding or perforation was reported. Among patients with complete pre- and post-procedural 24-h pH monitoring data, 73.7% showed a significant decrease in DeMeester score during follow-up. Another preliminary follow-up study (17) reported significant reductions in GERD-HRQL score, DeMeester score, and the fraction of time with esophageal pH < 4 at 3 and 6 months after treatment. More recently, a study of peroral endoscopic cardial constriction with band ligation in 68 patients with refractory GERD (15) showed significant improvement in reflux-related symptom scores at 12 months, with 77.9% of patients achieving complete independence from drug therapy and 76.5% reporting complete or partial satisfaction with symptom relief. Nonetheless, prospective evidence, particularly regarding device-related factors, remains insufficient.

At present, no randomized trial has evaluated ECCL performed using single-use endoscopes. We therefore designed a multicenter randomized controlled trial to compare single-use and reusable endoscopes for ECCL in patients with refractory GERD. The primary aim is to assess clinically significant symptom improvement, defined as a decrease in GERD-Q score of ≥4 points from baseline. Secondary aims include evaluating procedural feasibility, including image quality, maneuverability, and accessory compatibility, as well as safety outcomes, operational stability, device malfunction rates, and post-procedural acid-suppressive medication use.

## 2 Methods

### 2.1 Study design

This study is a multicenter, randomized, open-label, parallel-group controlled trial designed to compare the efficacy, safety, and procedural feasibility of endoscopic cardia constriction with ligation (ECCL) performed using a single-use endoscope versus a reusable endoscope in patients with refractory gastroesophageal reflux disease (GERD). Participants will be screened at the participating centers. Eligible patients will receive a full explanation of the study purpose, procedures, potential benefits, and possible risks from the investigators, and written informed consent will be obtained before enrollment.

**Figure 1:**
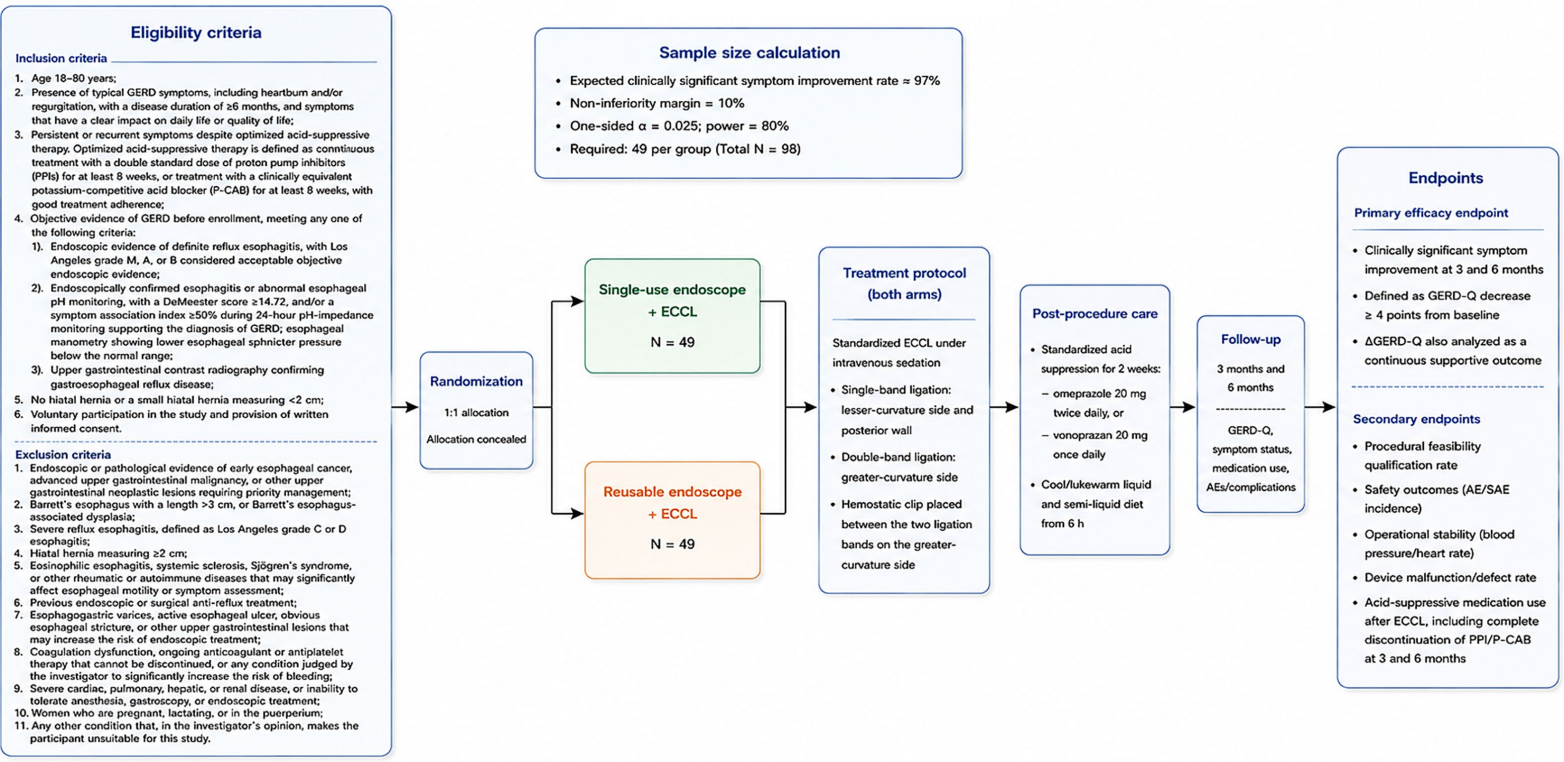
Flowchart of this trial.

After informed consent has been obtained and eligibility has been confirmed, participants will be randomly assigned in a 1:1 ratio to either the single-use endoscope ECCL group (intervention group) or the reusable endoscope ECCL group (control group). Data will be collected at baseline (screening/pre-procedure), during the procedure, and at follow-up visits at 3 and 6 months after ECCL through telephone/WeChat contact and/or outpatient visits (Figure 1).

This trial will use competitive enrollment across multiple sites, with each center recruiting participants according to local patient availability until the total target sample size is reached. Each site has a designated principal investigator responsible for local trial conduct and participant safety monitoring. The study will be conducted in accordance with the Declaration of Helsinki and has been approved by the Medical Ethics Committee of Shenzhen Hospital, Southern Medical University (Approval No. NYSZYYEC2025K055R001), the Ethics Committee of the 964th Hospital (approval no. 20250627 9), and the Ethics Committee of Daping Hospital (approval no. 2025271). The trial has been registered at ClinicalTrials.gov (registration no. NCT07176221).

### 2.2 Eligibility criteria

Participants will be enrolled according to the 2023 Chinese guidance of diagnosis and treatment of GERD and must meet the criteria for refractory GERD.

#### 2.2.1 Inclusion criteria

1 Age 18–80 years;
2 Presence of typical GERD symptoms, including heartburn and/or regurgitation, with a disease duration of ≥6 months, and symptoms that have a clear impact on daily life or quality of life;
3 Persistent or recurrent symptoms despite optimized acid-suppressive therapy. Optimized acid-suppressive therapy is defined as continuous treatment with a double standard dose of proton pump inhibitors (PPIs) for at least 8 weeks, or treatment with a clinically equivalent potassium-competitive acid blocker (P-CAB) for at least 8 weeks, with good treatment adherence;
4 Objective evidence of GERD before enrollment, meeting any one of the following criteria:
  1) Endoscopic evidence of definite reflux esophagitis, with Los Angeles grade M, A, or B considered acceptable objective endoscopic evidence;
  2) Endoscopically confirmed esophagitis or abnormal esophageal pH monitoring, with a DeMeester score ≥14.72, and/or a symptom association index ≥50% during 24-h pH-impedance monitoring supporting the diagnosis of GERD; esophageal manometry showing lower esophageal sphincter pressure below the normal range;
  3) Upper gastrointestinal contrast radiography confirming gastroesophageal reflux disease;
5 No hiatal hernia or a small hiatal hernia measuring <2 cm;
6 Voluntary participation in the study and provision of written informed consent.

#### 2.2.2 Exclusion criteria

1. Endoscopic or pathological evidence of early esophageal cancer, advanced upper gastrointestinal malignancy, or other upper gastrointestinal neoplastic lesions requiring priority management;
2. Barrett’s esophagus with a length >3 cm, or Barrett’s esophagus-associated dysplasia;
3. Severe reflux esophagitis, defined as Los Angeles grade C or D esophagitis;
4. Hiatal hernia measuring ≥2 cm;
5. Eosinophilic esophagitis, systemic sclerosis, Sjögren’s syndrome, or other rheumatic or autoimmune diseases that may significantly affect esophageal motility or symptom assessment;
6. Previous endoscopic or surgical anti-reflux treatment;
7. Esophagogastric varices, active esophageal ulcer, obvious esophageal stricture, or other upper gastrointestinal lesions that may increase the risk of endoscopic treatment;
8. Coagulation dysfunction, ongoing anticoagulant or antiplatelet therapy that cannot be discontinued, or any condition judged by the investigator to significantly increase the risk of bleeding;
9. Severe cardiac, pulmonary, hepatic, or renal disease, or inability to tolerate anesthesia, gastroscopy, or endoscopic treatment;
10. Women who are pregnant, lactating, or in the puerperium;
11. Any other condition that, in the investigator’s opinion, makes the participant unsuitable for this study.

### 2.3 Randomization, allocation concealment, and blinding

Eligible participants will be randomized in a 1:1 ratio to the intervention or control group using a computer-generated random number sequence. The randomization sequence will be generated by an independent statistician. Allocation will be concealed using sequentially numbered, opaque, sealed envelopes prepared by staff members who are not involved in participant recruitment or treatment. The envelopes will be opened only after written informed consent has been obtained and eligibility has been confirmed.

This is an open-label trial. Participants and endoscopists will be aware of group assignment because the type of endoscope used during the procedure cannot be masked. To minimize assessment bias, image-based evaluations and statistical analyses will be performed by medical personnel who are not involved in the intervention procedure.

### 2.4 Interventions and concomitant treatments

#### 2.4.1 ECCL procedure (both groups)

ECCL will be performed according to a standardized protocol in both groups. All procedures will be performed by experienced endoscopists who have independently performed at least 5,000 gastrointestinal endoscopic procedures and have received standardized training in the ECCL technique before trial initiation.

Before the procedure, all participants will be required to fast from food and water for at least 6 h. The procedure will be performed with the patient in the left lateral decubitus position under monitored intravenous sedation with propofol and ketamine. Vital signs, including blood pressure, electrocardiography, and oxygen saturation, will be continuously monitored throughout the procedure. Propofol will be administered at a maintenance dose of 6–12 mg/kg per hour, and ketamine will be administered at 1–2 mg/kg according to the anesthesiologist’s judgment and the patient’s clinical condition.

**Figure 2:**
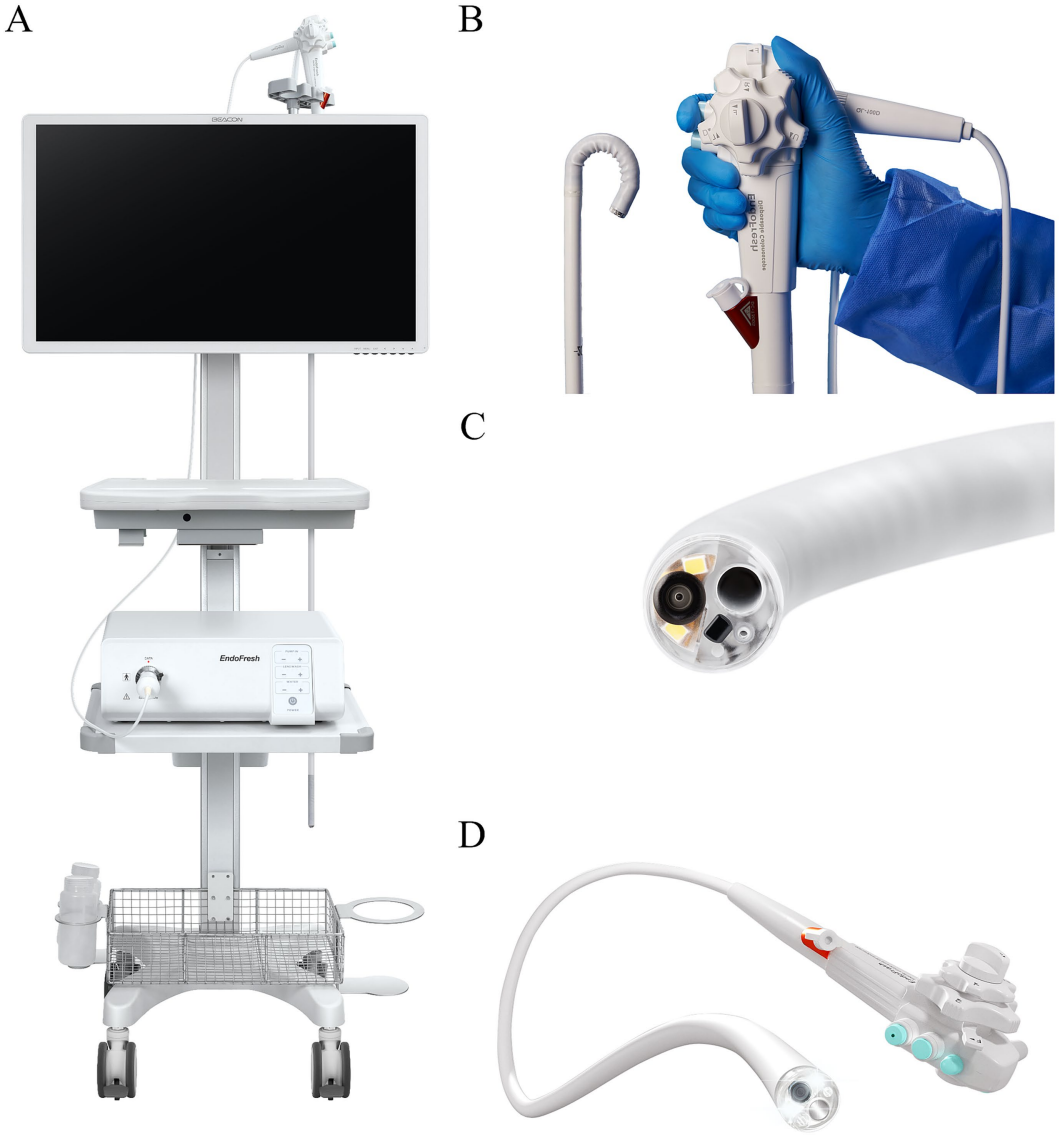
Single-use endoscope system used in the intervention group. **(A)** Overall view of the single-use endoscope system, including the main processor, display screen, and endoscope. **(B)** The endoscopist holds the control section of the single-use endoscope and operates the control knobs. **(C)** Close-up view of the distal tip of the single-use endoscope. **(D)** Full-length view of the single-use endoscope.

In the intervention group, ECCL will be performed using a single-use upper gastrointestinal endoscope (DG-T300S; EndoFresh, China) (Figure 2). In the control group, ECCL will be performed using a reusable flexible upper gastrointestinal endoscope with an outer diameter of 9.8 mm (EG-600WR; Fujifilm, Tokyo, Japan). In both groups, an endoscopic variceal ligation device (Speedband Superview Super 7; Boston Scientific Corporation, United States) will be mounted on the endoscope before ligation. A hemostatic clip (Resolution Clip; Boston Scientific Corporation, United States) will be used for fixation after ligation as specified in the protocol.

**Figure 3:**
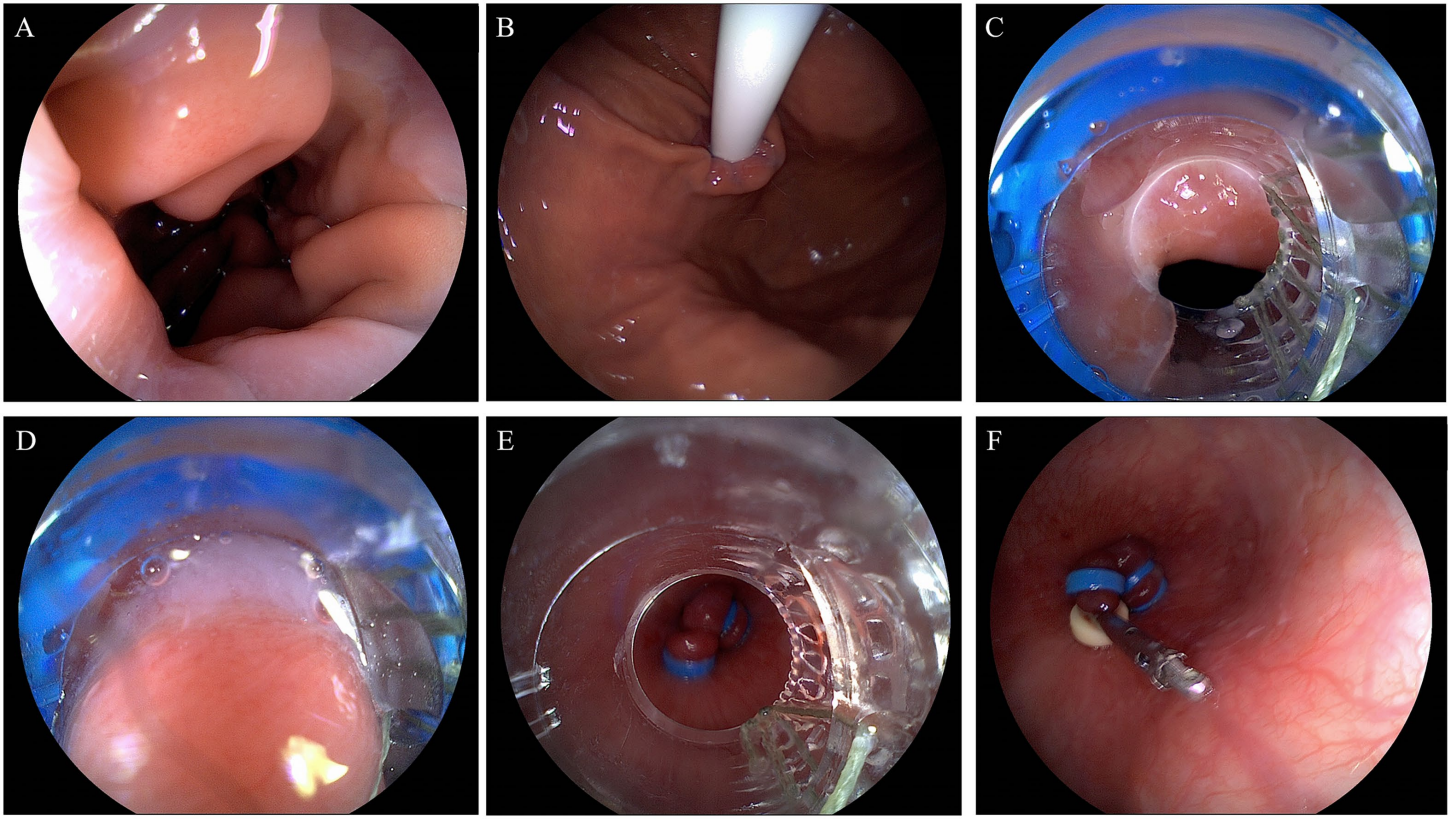
Standardized procedure of endoscopic cardia constriction ligation. **(A)** The esophagogastric junction is assessed in forward view. **(B)** The esophagogastric junction and cardia are further evaluated in retroflexed view. **(C)** After an endoscopic ligation device is mounted on the tip of the endoscope, the endoscope is advanced close to the target suction site. **(D)** Mucosal and underlying tissue is suctioned into the ligation device, followed by deployment of the ligation band. **(E)** The ligated sites are inspected after band deployment at the planned locations. **(F)** On the greater-curvature side, a hemostatic clip is placed between the two ligation bands, and the final post-ligation appearance of the cardia is assessed in forward view.

The ECCL procedure will be performed as follows (Figure 3). First, routine esophagogastroduodenoscopy will be performed to examine the esophagus, stomach, and duodenum and to exclude unexpected lesions that may affect the procedure. The esophagogastric junction will then be carefully assessed in both forward and retroflexed views. After adequate visualization of the cardia and confirmation of the target sites, an endoscopic variceal ligation device will be mounted on the tip of the endoscope. The endoscopist will then sequentially suction and ligate mucosal and underlying tissue at the lesser-curvature side of the cardia, the posterior wall, and the greater-curvature side of the cardia. Single-band ligation will be performed at the lesser-curvature side and posterior wall, whereas double-band ligation will be performed at the greater-curvature side. The target sites will be selected close to the esophagogastric junction according to the anatomical configuration of the cardia and the endoscopic view.

After completion of the three-point ligation, a hemostatic clip will be placed between the two ligation bands on the greater-curvature side to reduce the risk of band slippage and reinforce the constriction effect. The final appearance of the cardia will be reassessed in forward view, and the endoscopist will confirm whether the narrowing effect, visualization, and device performance are satisfactory.

During the procedure, investigators will document vital signs, including blood pressure, heart rate, electrocardiography, and oxygen saturation; procedural progress; procedure completion status; procedure time; intra-procedural image/video documentation; difficulty in retroflexion or visualization; suction performance; accessory compatibility, including ligator performance and stability; clip deployment; and any device-related issues, including image interruption, suction or water system blockage, leakage, or failure of accessory passage. Procedure-related adverse events, including mucosal injury, bleeding, suspected perforation, hemodynamic instability, and anesthesia-related events, will also be recorded in the electronic case report form.

#### 2.4.2 Post-procedure management

All participants will receive standardized short-term acid-suppressive therapy for 2 weeks after ECCL as part of routine post-procedural management. The recommended regimen will consist of either a proton pump inhibitor, such as omeprazole 20 mg twice daily, or a potassium-competitive acid blocker, such as vonoprazan 20 mg once daily, according to physician judgment, local availability, and patient tolerance. The medication class, drug name, dose, frequency, start and stop dates, adherence, and any medication changes will be systematically recorded. After completion of the scheduled 2-week post-procedural treatment, any continued use, discontinuation, dose reduction, or resumption of PPI/P-CAB therapy will be recorded at each follow-up visit and incorporated into the outcome analysis.

#### 2.4.3 Concomitant treatments

During the study period, the following concomitant treatments will be permitted under physician guidance when clinically indicated and will be documented in detail:

- Acid-suppressive therapy, including PPIs or P-CABs;
- Analgesics, such as acetaminophen;
- Prokinetic agents, such as mosapride or domperidone;
- Antibiotics, such as cephalosporins or quinolones.

Supportive measures, including probiotics, dietary adjustments, and psychological interventions for postoperative anxiety, will also be permitted and recorded to support interpretation of the study outcomes.

#### 2.4.4 Rescue management

If unexpected events occur, rescue treatment will be provided according to standard clinical practice. This may include vasoactive agents for hypotension, such as norepinephrine, ephedrine, or dopamine; hemostatic agents and/or blood transfusion for severe bleeding, such as desmopressin, thrombin, or blood products; endoscopic clip closure for small perforations and surgical management for larger perforations; airway support for respiratory depression, such as naloxone, endotracheal intubation, or mechanical ventilation; and guideline-based management for suspected myocardial infarction, such as aspirin, nitroglycerin, and cardiology intervention when needed. The rescue process, timing, treatments provided, monitoring data, and relevant imaging findings will be recorded for traceability.

### 2.5 Study endpoints

#### 2.5.1 Primary endpoint

The primary efficacy endpoint is clinically significant improvement in GERD symptoms, defined as a decrease in GERD-Q score of ≥4 points from baseline. The GERD-Q questionnaire has a score range of 0–18, with higher scores indicating more severe reflux symptoms. GERD-Q will be assessed at baseline before the procedure and at 3 and 6 months after ECCL. The proportion of participants achieving clinically significant symptom improvement will be compared between the two groups at each follow-up time point. In addition, the absolute change in GERD-Q score from baseline (ΔGERD-Q) will be analyzed as a continuous outcome.

#### 2.5.2 Secondary endpoints

Secondary endpoints include:

1 Procedural feasibility (clinical operability) assessed by:
  - Image quality (A: adequate brightness/contrast/clarity with clear identification of the dentate line; B: inadequate);
  - Maneuverability/retroflexion (A: successful retroflexion; B: restricted or failed);
  - Accessory compatibility (A: ligator matches well and works smoothly; B: mismatch or inability to complete);

A case is considered “qualified,” if all items are graded A; any grade B results in “unqualified.” The feasibility qualification rate will be calculated as qualified cases/total cases ×100%.

2 Safety outcomes, including procedure-related mucosal injury, perforation, and clinically significant bleeding occurring during ECCL or within 1 h after the procedure, as well as postoperative events, such as pain, delayed bleeding, and infection during follow-up. All adverse events (AEs) and serious adverse events (SAEs) will be recorded in the eCRF and classified. Safety outcomes will be summarized as incidence rates, calculated as: overall AE incidence (%) = (number of participants with ≥1 AE / total participants) × 100%, overall SAE incidence (%) = (number of participants with ≥1 SAE / total participants) × 100%, and for each specific complication (e.g., perforation, significant bleeding, infection), event-specific incidence (%) = (number of participants with the event / total participants) × 100%.
3 Operational stability, defined by the proportion of participants whose blood pressure and heart rate remain within physiological fluctuation ranges during the procedure (stability rate = number stable/total ×100%).
4 Device malfunction/defect rate, including intra-procedural technical failures, such as image interruption or suction/water system blockage/leakage (malfunction rate = cases with malfunction/total procedures ×100%).
5 Acid-suppressive medication use, including complete discontinuation, continuation, resumption, or dose/frequency reduction of PPI/P-CAB therapy at 3 and 6 months after ECCL. Complete discontinuation is defined as no use of any PPI or P-CAB at the corresponding follow-up time point. The PPI/P-CAB discontinuation rate will be calculated as: number of participants completely off PPI/P-CAB therapy / total participants × 100%. The proportions of participants requiring continued or resumed acid-suppressive therapy and those with reduced dose or frequency compared with baseline will also be summarized.

### 2.6 Sample size calculation

The sample size was determined based on a non-inferiority design. According to previous reports, the symptom relief rate after ECCL in patients with GERD is approximately 97%. Because both groups will undergo the same standardized ECCL procedure and differ only in the type of endoscope used, the expected clinically significant symptom improvement rate was assumed to be 97% in both groups. The non-inferiority margin was set at 0.10, representing the maximum clinically acceptable absolute reduction of 10% in the symptom improvement rate for the single-use endoscope group compared with the reusable endoscope group. With a one-sided *α* of 0.025, a power (1 − *β*) of 80%, and an anticipated dropout rate of 5%, 49 participants are required in each group, resulting in a total planned sample size of 98 patients. The sample size calculation was performed using R software, version 4.3.1.

### 2.7 Data collection

Data will be collected at baseline, during the procedure, and at follow-up time points at 3 and 6 months. Key variables include:

(1) Demographics: age, sex, and patient initials/code;
(2) Disease history and diagnostic information: duration of GERD symptoms, prior and current GERD medications, endoscopic findings relevant to eligibility, including LA grade and Hill grade, and objective diagnostic findings, including esophageal pH monitoring, 24-h pH-impedance monitoring, esophageal manometry, and upper gastrointestinal contrast radiography;
(3) Baseline assessments: physical examination and vital signs; laboratory tests, including blood routine, coagulation profile, and infection screening as required by the screening work-up; and pre-procedure ECG;
(4) Procedural data: assigned endoscope type, feasibility items, including image quality, maneuverability/retroflexion, and accessory compatibility, procedure completion status, intra-procedural image/video documentation, blood pressure and heart rate changes, and procedure-related complications;
(5) Device performance: device malfunction or defect events, such as image interruption, suction/water system blockage or leakage, and any device-related adverse events;
(6) Outcome measures: GERD-Q at baseline, 3 months, and 6 months; symptom changes and patient-reported recovery; postoperative complications; and any AEs/SAEs;
(7) Post-procedural acid-suppressive medication use: medication class, drug name, dose, frequency, treatment duration, adherence, discontinuation, dose reduction, and any additional or rescue acid-suppressive therapy during follow-up.

All participants will be followed for 6 months after ECCL. Follow-up assessments will be performed at 3 months and 6 months via telephone/WeChat contact and/or outpatient visits. GERD-Q scores, symptom status, patient-reported recovery, acid-suppressive medication use, and adverse events or complications will be recorded at each follow-up time point. Acid-suppressive medication assessment will include whether participants have discontinued, reduced, continued, or resumed PPI/P-CAB therapy after the scheduled 2-week post-procedural treatment. Participants reporting alarm symptoms, such as bleeding, fever, persistent or severe chest pain, progressive dysphagia, or other suspected complications, will be instructed to return to the hospital within 24 h for appropriate evaluation, including blood tests, chest CT, and/or endoscopy as clinically indicated.

### 2.8 Treatment termination

The study intervention may be temporarily suspended or terminated at the trial level, if serious safety concerns arise (e.g., perforation, major bleeding, severe infection) and the event rate exceeds a prespecified threshold, if major protocol deficiencies are identified, or if required by regulatory authorities. During any suspension period, outcome and safety data for enrolled participants will continue to be collected, and follow-up will proceed as planned. Trial resumption will require resolution of key safety/scientific issues and approval from the ethics committee and relevant regulatory bodies.

### 2.9 Withdrawal

Participants may withdraw from the study at any time. Reasons for withdrawal will be recorded whenever available, including withdrawal of consent, sponsor-initiated termination, serious adverse events affecting continuation, major protocol deviation, pregnancy, poor adherence, loss to follow-up, and concern about the safety of participants according to the investigators’ judgment.

### 2.10 Study schedule

The study schedule is organized into the three following phases: screening, treatment, and follow-up. The schedule is summarized in Table 1.

(1) Screening/baseline: informed consent; demographics and medical history; GERD symptom history; prior and current GERD medication use; eligibility assessment; objective GERD evidence assessment; physical examination and vital signs; laboratory tests, including blood routine, coagulation profile, and infection-related tests; ECG; baseline endoscopic assessment, including LA classification of reflux esophagitis and Hill grading for hiatal hernia as applicable; baseline GERD-Q.
(2) Treatment: randomization and allocated endoscope type; ECCL according to the standardized protocol; intravenous sedation record; intra-procedural vital signs; procedure time and completion status; feasibility items; intra-procedural image/video documentation; device performance and device-related problems; procedure-related adverse events; immediate safety check within 1 h after the procedure; standardized postoperative acid suppression for 2 weeks; diet advancement starting 6 h post-procedure.
(3) Follow-up: GERD-Q at 3 months and 6 months; symptom status; patient-reported recovery; acid-suppressive medication use, including discontinuation, continuation, dose reduction, resumption, and rescue PPI/P-CAB therapy; adverse events and complications; additional hospital evaluation if suspected complications or alarm symptoms occur.

**Table 1:** Study schedule.

Item	Screening/baseline	Procedure day	Immediate post-procedure	Follow-up 3 months	Follow-up 6 months
Written informed consent	✓				
Demographics and medical history	✓				
GERD symptom history	✓			✓	✓
Eligibility assessment	✓				
Objective GERD evidence assessment^a^	✓				
Baseline endoscopic assessment, including LA grade and Hill grade	✓				
Physical examination and vital signs	✓	✓	✓		
Laboratory tests^b^	✓				
ECG	✓				
Randomization and allocation	✓				
Assigned endoscope type		✓			
Anesthesia/sedation record		✓			
ECCL procedure		✓			
Intra-procedural image/video documentation		✓			
Procedural feasibility assessment^c^		✓			
Device performance assessment^d^		✓			
Procedure-related adverse events		✓	✓		
Immediate safety check within 1 h after procedure			✓		
Standardized post-procedural acid-suppressive therapy for 2 weeks		✓	✓		
GERD-Q questionnaire	✓			✓	✓
Symptom status and patient-reported recovery				✓	✓
Acid-suppressive medication use^e^	✓	✓		✓	✓
Adverse events and serious adverse events collection	✓	✓	✓	✓	✓
Assessment of complications or alarm symptoms		✓	✓	✓	✓

a Objective GERD evidence assessment refers to the objective diagnostic evidence required by the eligibility criteria.

b Laboratory tests include complete blood count, coagulation profile, and infection screening as required by the screening work-up.

c Procedural feasibility includes image quality, maneuverability/retroflexion, and accessory compatibility.

d Device performance includes image interruption, suction/water system blockage or leakage, failure of accessory passage, and other device-related problems.

e Acid-suppressive medication use includes medication class, drug name, dose, frequency, treatment duration, adherence, discontinuation, dose reduction, continuation, resumption, and any additional or rescue PPI/P-CAB therapy during follow-up.

### 2.11 Adverse events

All adverse events (AEs) occurring from the time of informed consent through the end of follow-up will be actively collected and recorded. Investigators will assess the likelihood that each AE is related to the ECCL procedure, the endoscope or device used (single-use or reusable), peri-procedural management, or concomitant medications, including acid suppressants, analgesics, prokinetics, and antibiotics.

AEs of special interest include, but are not limited to, mucosal injury, perforation, intra-procedural or post-procedural bleeding, including bleeding after band sloughing, infection, and post-procedural chest or retrosternal pain. Serious adverse events (SAEs) are defined as events that result in death, are life-threatening, require hospitalization or prolongation of hospitalization, lead to persistent or significant disability or incapacity, cause congenital anomaly or birth defect, or are considered other medically important events.

If an AE occurs, appropriate medical management will be provided according to clinical judgment and standard practice. This may include symptomatic treatment, endoscopic hemostasis, endoscopic closure, such as clipping for suspected perforation, blood transfusion when indicated, escalation to surgical or intensive care management for severe complications, and additional diagnostic evaluation as needed. For suspected SAEs, investigators will ensure immediate clinical intervention and complete the required reporting procedures within 24 h to the sponsor/applicant and relevant regulatory or ethics bodies, as specified in the protocol.

AE information will be documented in detail in the electronic case report form (eCRF), including onset and resolution dates, symptoms/signs or diagnosis, severity grading, causality assessment, actions taken, and outcomes. CTCAE v5.0 will be referenced for severity grading when applicable. Participants with ongoing AEs at study completion will continue to be followed until the event resolves or stabilizes.

### 2.12 Protocol deviations and violations

Protocol deviations will be documented throughout the study.

A minor protocol deviation is defined as a departure from the protocol that is unlikely to affect participant safety or the assessment of study outcomes, such as a follow-up contact occurring slightly outside the scheduled visit window without affecting endpoint collection.

A major protocol violation is defined as a deviation that may compromise participant safety, study integrity, or endpoint validity. Examples include failure to obtain valid informed consent, enrollment of participants who do not meet the eligibility criteria, incorrect randomization or breach of allocation concealment, unreported SAEs, use of prohibited medications that may materially affect outcomes, failure to complete the assigned intervention due to protocol-related errors, or missing multiple follow-up assessments that preclude evaluation of the primary endpoint. Major protocol violations will be reviewed by the study leadership and may lead to exclusion from the per-protocol analysis, while participants with such violations will remain included in the intention-to-treat (ITT) analysis.

### 2.13 Data analysis

The primary analysis will follow the intention-to-treat (ITT) principle, including all randomized participants analyzed according to their assigned group. The per-protocol (PP) analysis set will include participants who receive the allocated intervention and complete the key follow-up assessments without major protocol violations. The safety set will include all participants who undergo ECCL and have at least one post-procedure safety assessment.

Descriptive statistics will be used to summarize baseline characteristics and outcomes. Continuous variables will be presented as mean ± standard deviation or median with range, depending on data distribution. Categorical variables will be summarized as counts and percentages. Between-group comparisons will be performed using the chi-square test or Fisher’s exact test for categorical variables and the t test or Wilcoxon rank-sum test for continuous variables, as appropriate.

The primary efficacy endpoint is clinically significant symptom improvement, defined as a decrease in GERD-Q score of ≥4 points from baseline. For the non-inferiority analysis, the between-group difference in clinically significant symptom improvement rates will be calculated as the rate in the single-use endoscope group minus the rate in the reusable endoscope group, together with the corresponding 95% confidence interval. Non-inferiority will be concluded if the lower bound of the 95% confidence interval for the between-group difference is greater than −0.10. The primary non-inferiority analysis will be performed in the ITT population, with the PP analysis used as a supportive analysis.

The absolute change in GERD-Q score from baseline (ΔGERD-Q) at 3 and 6 months will be analyzed as a continuous supportive outcome. Secondary analyses will compare procedural feasibility qualification rates, operational stability rates, device malfunction or defect rates, and AE/SAE incidence between the two groups.

Post-procedural acid-suppressive medication use will be systematically incorporated into the analysis because it may influence symptom-related outcomes. The scheduled 2-week post-procedural acid-suppressive therapy will be summarized by medication type, dose, frequency, treatment duration, and adherence. After completion of the scheduled 2-week treatment, PPI/P-CAB use at 3 and 6 months will be compared between groups, including complete discontinuation, continuation, resumption, dose reduction, and additional or rescue acid-suppressive therapy. The proportion of participants who are completely off PPI/P-CAB therapy at 3 and 6 months will be analyzed as a secondary endpoint.

Sensitivity analyses will be performed to assess the robustness of the primary outcome by adjusting for post-procedural acid-suppressive medication use, including medication type, adherence, and continuation or resumption of PPI/P-CAB therapy after the scheduled 2-week treatment. If appropriate, additional sensitivity analyses may be performed after excluding participants with major deviations in post-procedural acid-suppressive therapy.

For non-inferiority testing, a one-sided significance level of 0.025 will be used. For secondary and exploratory analyses, a two-sided *p* value <0.05 will be considered statistically significant. Statistical analyses will be performed using SPSS version 26.0.

## 3 Discussion

This multicenter randomized controlled trial is designed to compare the clinical efficacy, safety, and procedural feasibility of endoscopic cardia constriction with ligation (ECCL) performed using a single-use endoscope versus a conventional reusable endoscope in patients with refractory gastroesophageal reflux disease (GERD). By integrating patient-reported symptom outcomes with standardized assessments of procedural performance and device-related events, this study aims to generate prospective evidence relevant to both therapeutic endoscopy and the evolving role of single-use endoscopic technology.

Refractory GERD represents a clinically important subgroup of patients who continue to experience symptoms despite optimized acid-suppressive therapy (18). For these patients, long-term pharmacologic treatment may be associated with safety concerns, incomplete symptom control, and impaired quality of life, underscoring the need for effective minimally invasive alternatives (19). ECCL has emerged as a technically straightforward endoscopic intervention that may alleviate reflux by mechanically narrowing the cardia and enhancing lower esophageal sphincter competence through tissue remodeling (17, 20). Although encouraging outcomes have been reported, most available data are derived from non-randomized or single-center studies, and prospective comparative evidence remains limited. Existing clinical studies (15–17) suggest that ECCL-related techniques may improve both subjective symptoms and objective reflux parameters. Reported benefits include reductions in GERD-HRQL scores, DeMeester scores, and esophageal acid exposure, as well as a substantial proportion of patients achieving independence from acid-suppressive therapy after treatment. These findings support the feasibility of ECCL as an endoscopic anti-reflux approach, while also highlighting the need for rigorously designed prospective trials.

Several other endoscopic anti-reflux techniques have also been developed, including anti-reflux mucosectomy (ARMS), anti-reflux mucosal ablation (ARMA), and related modified approaches, such as ARMS with additional myoplasty or valve reconstruction. These procedures aim to remodel the gastroesophageal junction through mucosal resection, ablation, or scar formation and may provide a more standardized mucosal defect than ligation-based techniques. A systematic review and meta-analysis of ARMS reported high technical success and favorable clinical response, with significant improvements in GERD-related quality of life, GERD-Q score, and acid exposure time (21). However, adverse events, including dysphagia, esophageal stricture, and bleeding, have also been reported, and some studies of ARMS have described perforation as a potential serious complication (22, 23). ARMA was developed partly to simplify scar-inducing therapy and reduce the risks associated with mucosal resection. Emerging clinical evidence, including randomized studies, has supported its efficacy and safety in selected patients with GERD, although direct comparative evidence among ARMA, ARMS, ECCL, and other endoscopic anti-reflux techniques remains insufficient (24). Therefore, these techniques should be viewed as complementary rather than directly competing approaches, each with distinct technical requirements, risk profiles, and target populations.

Single-use endoscopes have been introduced primarily to address infection-control and reprocessing-related challenges associated with reusable devices (25). Prior investigations have suggested that single-use endoscopes can provide acceptable imaging quality and maneuverability for diagnostic procedures and selected therapeutic applications (26, 27). However, ECCL imposes specific technical requirements, including stable visualization, reliable suction, retroflexion, and seamless compatibility with ligation accessories. For this reason, ECCL provides a practical procedural model for evaluating whether a single-use endoscope can support therapeutic upper gastrointestinal interventions beyond routine diagnostic examination. Importantly, the present study is not designed to compare ECCL with ARMS, ARMA, or other endoscopic anti-reflux procedures. Instead, it focuses on whether ECCL performed using a single-use endoscope can achieve clinical efficacy, safety, and procedural feasibility comparable to ECCL performed using a reusable endoscope. This trial therefore directly evaluates whether the technical demands of ECCL can be met using single-use endoscopes without compromising clinical outcomes or safety, an issue that has not been systematically examined in previous studies.

The primary endpoint of clinically significant symptom improvement assessed by the GERD-Q questionnaire reflects a patient-centered approach that is consistent with routine clinical practice. GERD-Q is a validated instrument that captures both symptom frequency and symptom-related impact, and the use of a predefined threshold for clinically meaningful improvement facilitates interpretation and comparison with existing literature (28). Assessment at both 3 and 6 months will allow evaluation of short- to mid-term symptom control after ECCL and provide insight into the temporal pattern of treatment response across endoscope types.

In addition to efficacy, this study places particular emphasis on procedural feasibility and safety. By prospectively defining and recording parameters such as image quality, maneuverability, accessory compatibility, hemodynamic stability, device malfunction rates, and procedure-related adverse events, the trial provides a comprehensive evaluation of endoscope performance in a therapeutic setting. This approach extends beyond traditional efficacy endpoints and may provide practical information relevant to endoscopist training, device selection, and institutional adoption strategies.

The multicenter design enhances the generalizability of the findings and reflects real-world practice across different clinical settings. Standardization of the ECCL procedure, peri-procedural management, and outcome assessment may reduce operator-related variability and support the internal validity of the comparisons. Collectively, these design features position the study to provide meaningful evidence regarding both the clinical role of ECCL in refractory GERD and the applicability of single-use endoscopes in therapeutic upper gastrointestinal interventions.

Several limitations should be acknowledged. First, ECCL remains an emerging endoscopic anti-reflux technique, and the available evidence is still derived mainly from preliminary studies, retrospective analyses, and relatively small cohorts. Second, the present study does not compare ECCL with other endoscopic anti-reflux procedures, such as ARMS, ARMA, or ARMP/V; therefore, the results should not be interpreted as evidence that ECCL is superior or equivalent to these alternative techniques. Third, the selected study population excludes patients with severe reflux esophagitis, long-segment Barrett’s esophagus, or large hiatal hernia, and the findings may not be generalizable to these more complex GERD populations. Fourth, although single-use endoscopes may be most attractive in high-infectious-risk or medically fragile patients, such populations are excluded from the present trial for ethical and safety reasons. Therefore, this study should be regarded as an initial evaluation of whether a single-use endoscope can safely and effectively support ECCL in a relatively stable refractory GERD population. If safety, efficacy, and procedural feasibility are established, future studies may extend the evaluation to vulnerable or high-infectious-risk populations and incorporate formal cost-effectiveness analyses. Nevertheless, by using a randomized multicenter design and standardized procedural assessment, this trial may provide useful evidence regarding the feasibility of single-use endoscopes in ECCL and help inform future comparative studies of endoscopic anti-reflux therapies.

In summary, this trial is designed to clarify whether ECCL performed with a single-use endoscope can achieve symptom improvement, safety, and procedural performance comparable to those achieved with a reusable endoscope. The findings may provide evidence to support future clinical decision-making and further research on the integration of single-use endoscopic devices into therapeutic endoscopy for refractory GERD.
